# Insights in Osteosarcoma by Proton Nuclear Magnetic Resonance Serum Metabonomics

**DOI:** 10.3389/fonc.2020.506959

**Published:** 2020-10-16

**Authors:** Melissa Quintero Escobar, Tássia Brena Barroso Carneiro Costa, Lucas G. Martins, Silvia S. Costa, André vanHelvoort Lengert, Érica Boldrini, Sandra Regina Morini da Silva, Luiz Fernando Lopes, Daniel Onofre Vidal, Ana C. V. Krepischi, Mariana Maschietto, Ljubica Tasic

**Affiliations:** ^1^Department of Organic Chemistry, Institute of Chemistry, University of Campinas (UNICAMP), Campinas, Brazil; ^2^Facultad de Ingeniería Industrial, Universidad de Lima, Lima, Peru; ^3^Department of Genetics and Evolutionary Biology, Human Genome and Stem-Cell Research Center (CEGH-CEL), Institute of Biosciences, University of São Paulo (USP), São Paulo, Brazil; ^4^Molecular Oncology Research Center (CPOM), Barretos Cancer Hospital, Barretos, Brazil; ^5^Barretos Children's Cancer Hospital, Barretos, Brazil; ^6^Department of Pathology, Barretos Cancer Hospital, Barretos, Brazil; ^7^Brazilian Biosciences National Laboratory (LNBio), Brazilian Center for Research in Energy and Materials (CNPEM), Campinas, Brazil

**Keywords:** lipid alterations, NMR, metabonomics, osteosarcoma, bone cancer, pediatric cancers

## Abstract

Pediatric osteosarcoma outcomes have improved over the last decades; however, patients who do not achieve a full resection of the tumor, even after aggressive chemotherapy, have the worst prognosis. At a genetic level, osteosarcoma presents many alterations, but there is scarce information on alterations at metabolomic levels. Therefore, an untargeted nuclear magnetic resonance metabonomic approach was used to reveal blood serum alterations, when samples were taken from 21 patients with osteosarcoma aged from 12–20 (18, 86%) to 43 (3, 14%) years before any anticancer therapy were collected. The results showed that metabolites differed greatly between osteosarcoma and healthy control serum samples, especially in lipids, aromatic amino acids (phenylalanine and tyrosine), and histidine concentrations. Besides, most of the loading plots point to protons of the fatty acyls (-CH_3_ and -CH_2_-) from very-low- and low-density lipoproteins and cholesterol, as crucial metabolites for discrimination of the patients with osteosarcoma from the healthy samples. The relevance of blood lipids in osteosarcoma was highlighted when analyzed together with the somatic mutations disclosed in tumor samples from the same cohort of patients, where six genes linked to the cholesterol metabolism were found being altered too. The high consistency of the discrimination between osteosarcoma and healthy control blood serum suggests that nuclear magnetic resonance could be successfully applied for osteosarcoma diagnostic and prognostic purposes, which could ameliorate the clinical efficacy of therapy.

## Introduction

Despite being rare, cancer is the disease that causes the most death in children and adolescents in developed countries. However, with early treatment, it is highly curable (1). Among the pediatric cancers, osteosarcoma is the commonest primary malignant bone tumor developing during periods of rapid growth (2), with an event-free survival of 60 to 70% in 3 years. Between 10 and 20% of the patients have metastases at diagnosis, whereas ~40% will develop metastasis during treatment (3). Currently, the diagnosis is dependent on the clinical signs, radiographic assessment, and pathological stage, and surgery and intensive multi-agent chemotherapy are commonly used for treatment (4). Although there were many advances in the field, which contributed to reduced morbidities associated with treatment and management of osteosarcoma over the past few decades (5), no substantial improvement has been achieved in survival rate (6). Patients with metastatic disease and/or recurrence after treatment continue to have a poor prognosis, with a relapse rate of up to 35% (7, 8).

Osteosarcomas present several genomic alterations including chromosomal rearrangements, amplifications, point mutations, loss of heterozygosity, as well as epigenetic abnormalities, such as hypermethylation of tumor suppressor genes (9–11). Genomic analyses pointed out that osteosarcoma genesis mostly resides in early chromosomal changes. Yet, it is still unclear how these events start (5). These reports detailed the patterns of DNA copy number alterations, suggesting that these chromosomal rearrangements could amplify the oncogenes and cause loss of tumor suppressors, leading to tumor progression (5). In this scenario, the metabonomic analysis can provide a better understanding of the global biochemical dysregulation of osteosarcomas (that could ameliorate the clinical efficacy of therapy) and reveal qualitative and quantitative final products of cellular regulatory pathways (12). In osteosarcoma, changes in gene expression of *ENO1, TPI1, PKG1*, and *LDHC* compared with normal osteoblastic cell lines pointed to alterations in the glycolysis pathway (7). Additionally, alterations in arginine, glutathione, fatty acids (FAs), and *myo*-inositol were reported (13), as well as changes in the metastatic phenotype associated with modifications in cellular respiration, lactate production, and oxygen consumption (14, 15). Although these data contribute to the understanding of the biochemical pathways' alterations in osteosarcoma, molecular basis and metabonomics information are still scarce. Therefore, our research brings nuclear magnetic resonance (NMR) metabonomics on blood serum samples from mostly young patients with osteosarcoma and aims to potentially disclose biomarkers that might be useful in clinical applications.

## Methods

### Characteristics of the Osteosarcoma Cohort

Twenty-one blood serum samples of patients with osteosarcoma stored in the biobank of Barretos Cancer Hospital (SP, Brazil) were selected. The patients were diagnosed with osteosarcoma between 2008 and 2014. Eighteen patients presented the complete clinical and pathological information, as depicted in Table 1. Blood samples collected from eight healthy individuals were used as a control group (five females and three males), six of them from 4 to 9 years old and two adults with 32 and 36 years old, respectively. The serum samples were centrifuged at 3,939 g for 15 min at 4°C and stored at −80°C until NMR analysis.

**Table 1:** Clinical summary of patients with osteosarcoma.

**Case no. **	**Sex **	**Age (years) **	**Histologic type **	**Huvos grade **	**Meta (diag) **	**Status **	**Weight at diagnosis (kg) **
1	M	17	Telangectatic	NR	NR	Alive without disease	57
2	F	18	Osteoblastic	II	Absent	Death due to cancer	36
3	M	35	Osteoblastic	I	Present	Death due to cancer	74.1
4	M	20	Osteoblastic	I	Absent	Death due to cancer	65
5	M	14	Chondroblastic	I	Absent	Death due to cancer	57.2
6	F	15	Osteoblastic	I	Present	Death due to another disease (sepsis)	52.0
7	M	17	Osteoblastic	II	Absent	Death due to cancer	65.3
8	M	43	Osteoblastic	II	Absent	Death due to cancer	69.9
9	M	17	Osteoblastic	I	Present	Death due to cancer	67.5
10	F	17	Fibroblastic	I	Absent	Alive without disease	50.7
11	F	18	Osteoblastic	II	Present	Death due to cancer	55.5
12	F	12	Osteoblastic	II	Present	Death due to cancer	39.1
13	M	13	NR	NR	Present	Alive without disease	72.6
14	F	19	Osteoblastic	II	Present	Death due to cancer	73.7
15	F	16	Telangectatic	I	Present	Death due to another disease (sepsis)	51.5
16	F	29	Osteoblastic	I	Absent	Death due to cancer	NR
17	F	19	Parosteal	NR	Absent	Alive without disease	45.5
18	M	12	Osteoblastic	I	Absent	Alive without disease	62.8
19	M	4	NI	NI	NI	NI	NI
20	M	9	NI	NI	NI	NI	NI
21	F	4	NI	NI	NI	NI	NI

*Number: all samples were duplicated and were treated like different samples. NR, non-reported data; NI, not informed or without written consent to disclose data*.

### Nuclear Magnetic Resonance Spectroscopy Analyses

For NMR analyses, 250 μl of each serum sample and 250 μl of deuterium oxide solvent (99.9% deuterium oxide with 0.03% of trimethylsilyl propanoic acid, Sigma Aldrich) were transferred to 5-mm NMR tubes. All the samples were duplicated, except two controls, and treated like different samples. The ^1^H-NMR spectra were acquired at 25°C using a 600-MHz NMR spectrometer Bruker AVANCE III (Bruker Biospin, Karlsruhe, Germany), 600.13 MHz for ^1^H frequency, equipped with a triple resonance broadband inverse probe. One-dimensional proton NMR spectra with water suppression were recorded using the water suppression by gradient tailored excitation (WATERGATE) pulse sequence—p3919g—with 128 scans. ^1^H-NMR spectra with T_2_ filters were acquired using the Carr–Purcell–Meiboom–Gill (CPMG) pulse sequence (*cpmgpr1d*), also with 128 scans. The two-dimensional total correlation spectroscopy (mlevphpr.2) experiments were performed, with 256 scans, to randomly selected samples.

### Data Analysis: Nuclear Magnetic Resonance Data Processing and Statistics

All serum ^1^H-NMR spectra were manually processed (phase and baseline corrections) using MestReNova software (9.0.1-13254). Trimethylsilyl propanoic acid (0.00 ppm) was the chemical shift reference. The spectra were divided into regions with equal width of 0.005 ppm, called bins, and used to construct the matrix for multivariate analysis. The picks of HDO (δ 4.70–5.00), ethylenediaminetetraacetic acid (δ 2.53–2.73, δ 3.06–3.28, and δ 3.6–3.65), and ethanol (δ 1.16–1.22 and δ 3.58–3.68, from antiseptic swabbing at sample collection) were excluded from analysis (Supplementary Figures S1, S2). Samples were normalized to a constant sum (100) of all spectra intensity to reduce the concentration differences. The metabolites were assigned based on chemical shifts, coupling constants, and databases—Human Metabolome Database and BioMagResBank (16, 17).

### Multivariate Data Analyses

The multivariate analyses were performed using the MATLAB software (v. R2015a, Mathworks Inc) and the MetaboAnalyst platform (18). The matrices were constructed with 56 spectra (2 × 21 = 42 for patients with osteosarcoma, and 2 × 6 + 2 = 14 healthy controls) and 1,567 or 1,489 variables for CPMG and WATERGATE spectra, respectively. Two principal component analyses (PCAs) were conducted. The first one using all spectra to identify outliers and explore natural grouping within samples. The second was applied to patients' spectra to investigate the influence of clinical characteristics on their metabolome.

Partial least squares discriminant analysis (PLS-DA) models were constructed to classify the samples from patients with osteosarcoma or healthy controls, using the classification toolbox for MATLAB functions (19). The models were validated using leave-one-out cross-validation (LOOCV). Variable importance in projection (VIP) was analyzed to depict the most different chemical shifts among analyzed samples. Confusion matrices were constructed. Accuracy, specificity, and sensitivity values were computed (20).

The different metabolites between the two groups were identified by the assignment of the spectral bins with the highest VIP values in the PLS-DA model. A receiver operating characteristic curve analysis was performed based on linear support vector machines and using Monte Carlo cross-validation, in which two-thirds of the samples were used to build the classification models followed by validation on the one-third that was previously left out, to evaluate the predictive capacity of the picked metabolites.

As previously described (21), PCA and PLS-DA were applied to the aromatic region of the ^1^H-NMR CPMG spectra, δ 6.50–9.00 (56 samples and 501 variables), to evaluate the influence of some metabolites that are present in serum in low concentrations.

Univariate statistical analysis was also performed to evaluate the difference of the discriminant metabolites, identified by the PLS-DA analyses, between osteosarcoma patients and healthy controls. *T*-test was applied using MetaboAnalyst, and *P* < 0.05 was considered a statistically significant difference.

## Results

### Proton Nuclear Magnetic Resonance Data of Serum Samples in Osteosarcoma Patients

**Figure 1:**
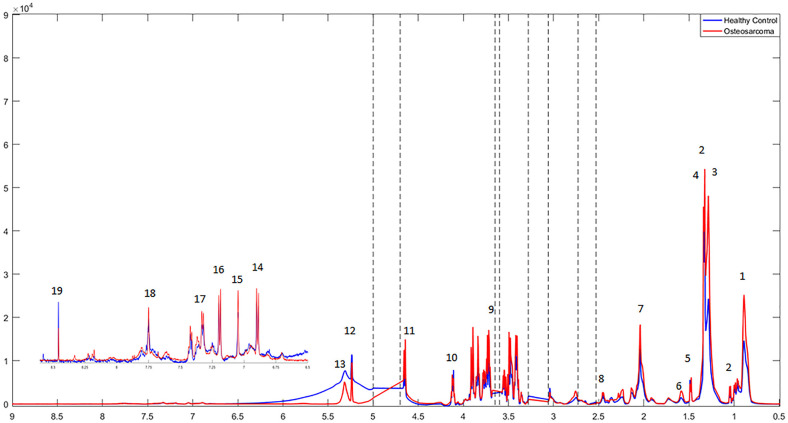
^1^H-NMR mean spectra of osteosarcoma patients (in red) and healthy controls (in blue) acquired using CPMG (*cpmgpr1d*) pulse sequence. Main signals have been assigned: **1**. Fatty acyls' -CH_3_; **2**. Valine (Val); **3**. Fatty acyls' -CH_2_- hydrogens as in CH_3_(CH_2_)_n_; **4**. Lactate (Lac); **5**. Alanine (Ala); **6**. Fatty acyls' from -CH_2_- group next to carboxyl group as in CH_2_CH_2_C(O); **7**. Fatty acyls' CH_2_CH=; **8**. Glutamine (Gln); **9**. Glucose (Glc); (*glucose resonance hydrogens); **10**. Fatty acyls' -CH=CH-; **11**. tyrosine (Tyr); **12**. histidine (His); **13**. phenylalanine (Phe); **14**. formate (For). Excluded spectral regions are indicated with the dotted lines.

The ^1^H-NMR mean spectra revealed some differences between patients with osteosarcoma and healthy controls, in the regions of δ 0.85–0.89 (m), δ 1.24–1.37 (m), δ 1.55–1.65 (m), δ 1.98–2.09 (m), and δ 5.29–5.43 (m). These regions refer to fatty acyls' methyl group hydrogens -C**H**_3_,–fatty acyls' -C**H**_2_-, hydrogens as in CH_3_(C**H**_2_)_n_-, fatty acyls' from -CH_2_- group next to carboxyl group as in CH_2_C**H**_2_C(O), fatty acyls' CH_2_C**H**=, and fatty acyls' CH=C**H**-. Also, the peaks from the region δ 6.00–δ 8.00 ppm were also altered in patients with osteosarcoma and attributed to aromatic amino acids—tyrosine (Tyr), phenylalanine (Phe), and histidine (His) (Figure 1, Supplementary Table S1).

### Multivariate Statistical Analysis

**Figure 2:**
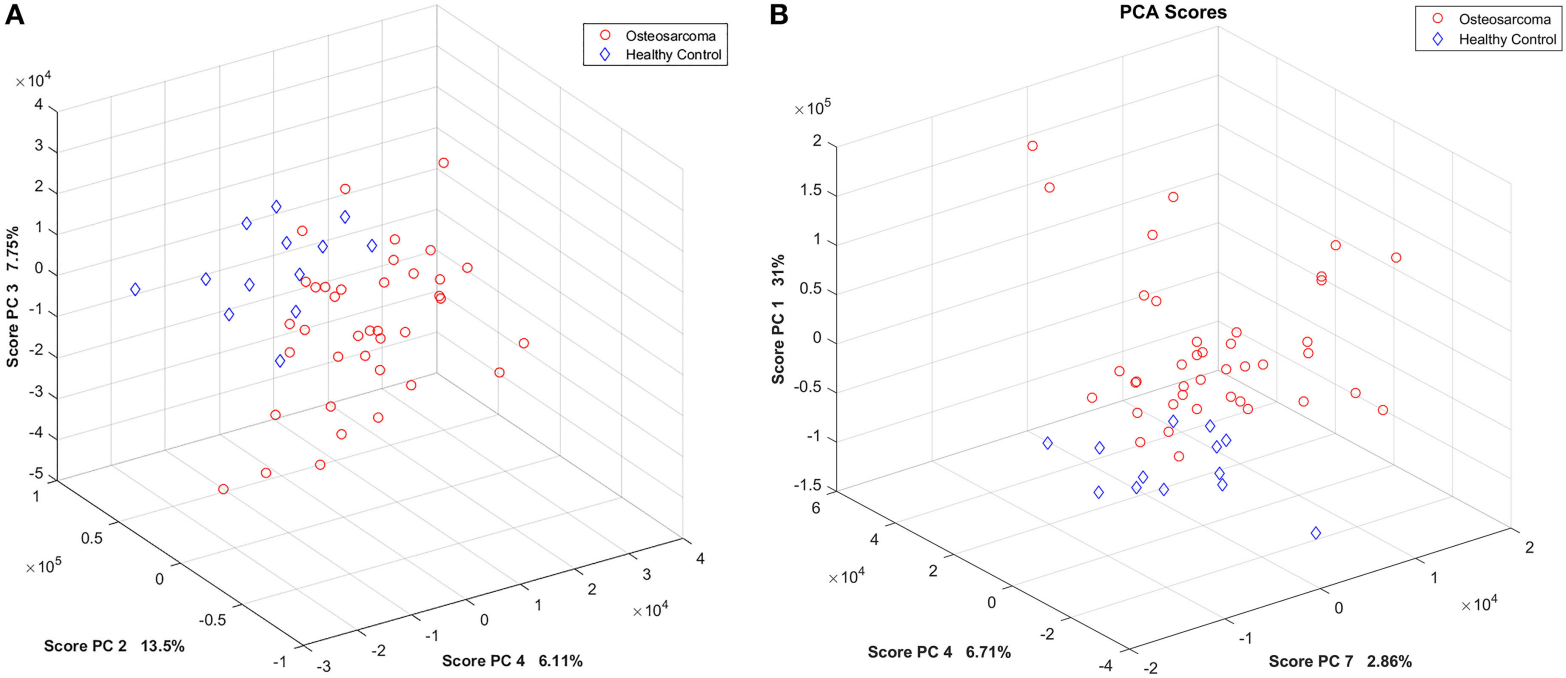
PCA score plots. **(A)** PCs 2, 3, and 4 of the ^1^H-NMR CPMG spectra model. **(B)** PCs 1, 4, and 7 of the ^1^H-NMR WATERGATE spectra model. Patients with osteosarcoma are shown in red circles, and healthy controls are shown in blue diamonds.

An exploratory analysis based on principal components (PCs) revealed five and three outliers on CMPG and WATERGATE spectral data, respectively (Supplementary Figures S3, S4). The outliers were removed from the dataset before further analyses. Supplementary Figures S5, S6 show the scores' plots of the first three PCs of the CPMG and WATERGATE spectra PCA models. In the CPMG spectra model, a tendency of sample clustering related to patients with osteosarcoma and healthy controls was observed on the PCs 2, 3, and 4, explaining 27.4% of the samples' variance (Figure 2A). For the WATERGATE spectra model, clustering in cancer and control groups was seen on PCs 1, 4, and 7, which explain 40.6% of the variance (Figure 2B). Supplementary Figures S7, S8 contain their corresponding loading plots.

**Figure 3:**
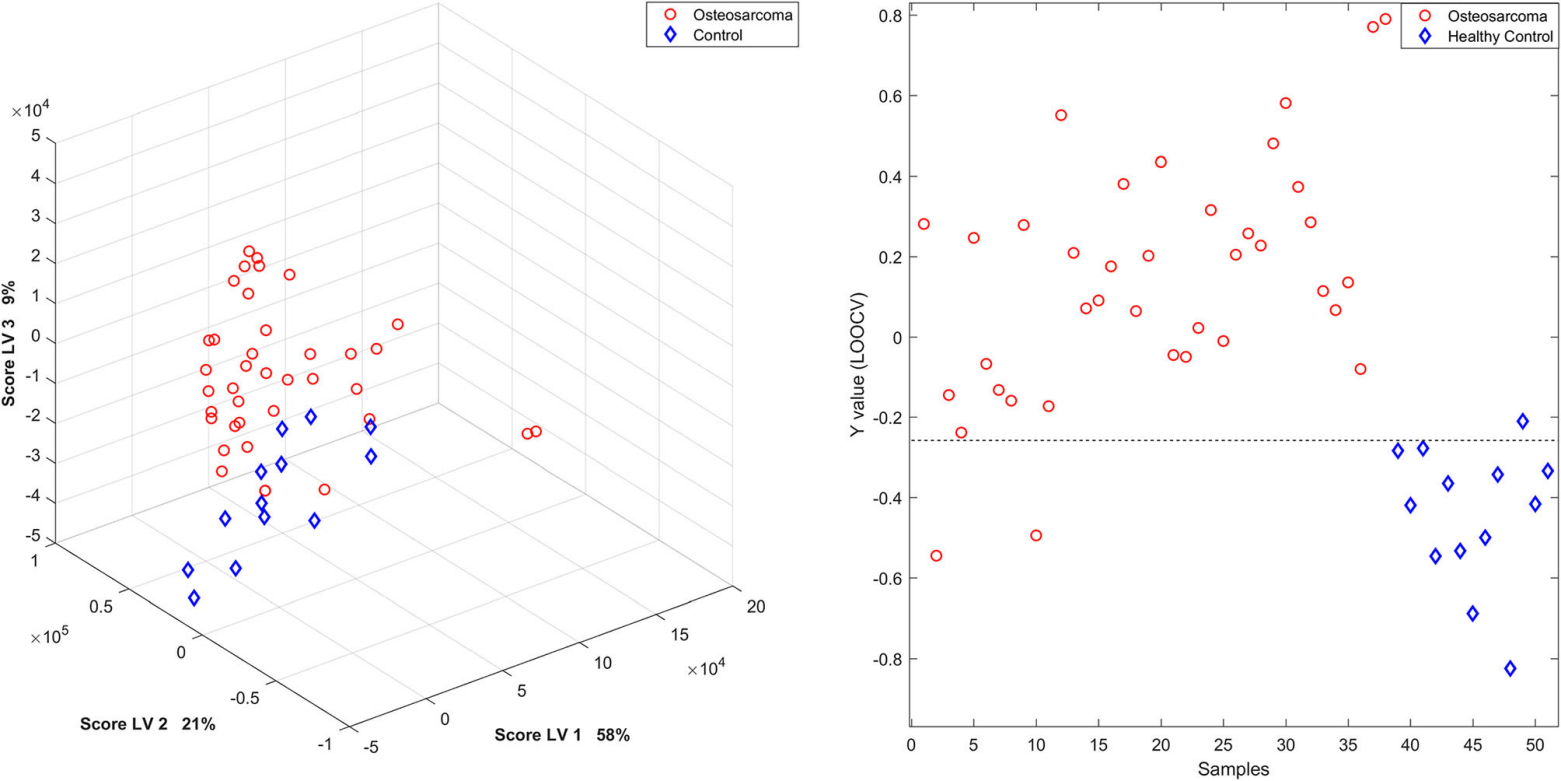
PLS-DA scores' plot and Y calculated for each sample, by LOOCV, of the ^1^H-NMR serum samples in the CPMG spectra model. Patients with osteosarcoma are shown in red circles and healthy controls in blue diamonds.

**Figure 4:**
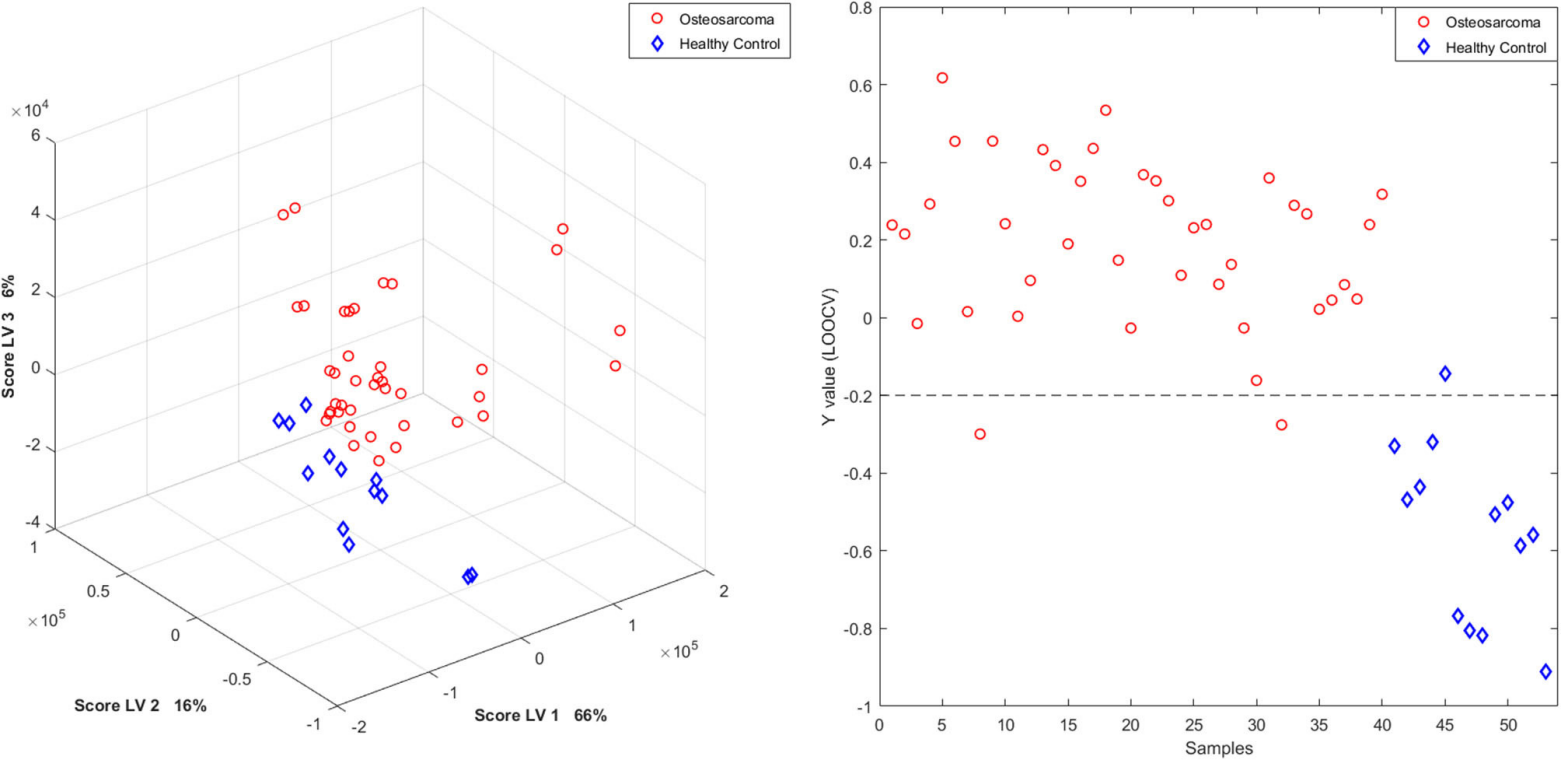
PLS-DA scores plot and Y calculated for each sample, by LOOCV, of the ^1^H-NMR serum samples recorded with Watergate. Patients with osteosarcoma are shown in red circles and healthy controls in blue diamonds.

Additionally, the built PLS-DA model separated cancer patients from healthy controls (Figures 3, 4), and, by LOOCV, the ^1^H-NMR CPMG, and WATERGATE spectra models reached 92.2 and 94.3% of accuracy applying four and six latent variables, respectively (Supplementary Table S2).

**Figure 5:**
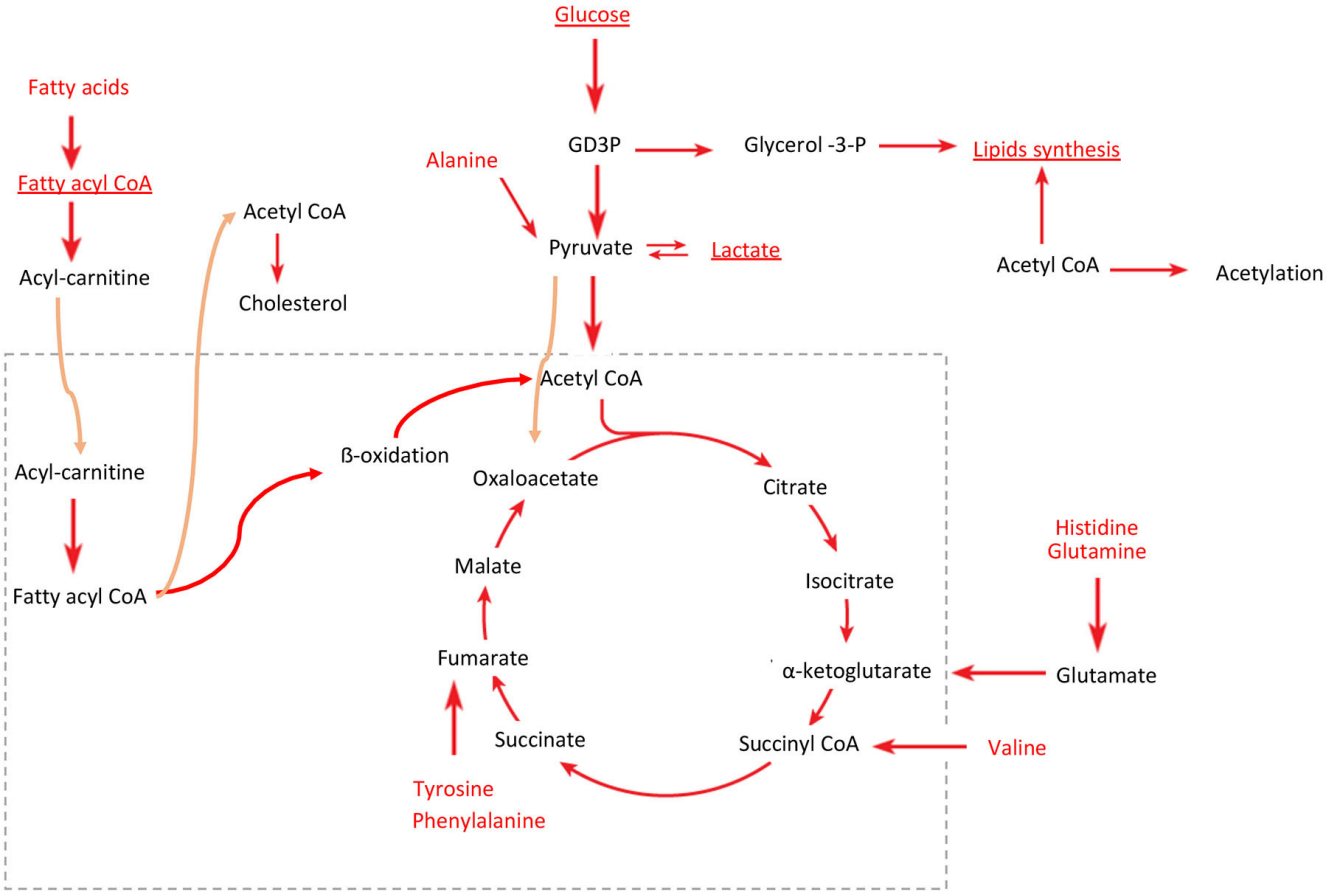
Predicted perturbed energy metabolism pathways in osteosarcoma. Red indicates the metabolites identified by ^1^H-NMR. Underlined are the key metabolites that discriminated against the patients with osteosarcoma from healthy controls in the PLS-DA model, identified by the higher VIP scores. Dotted box represents mitochondria, and orange arrows indicate the metabolite flux between the inside and outside of the mitochondria.

Lactate, FAs, and glucose were altered in the serum of patients (Figure 5), contributing to the separation between osteosarcoma samples and healthy controls. These metabolites also presented statistically significant differences between osteosarcoma patients and healthy controls in univariate analysis (Supplementary Figure S9). The metabolite assignments are shown in Supplementary Table S1 and Supplementary Figure S10. The receiver operating characteristic curve presented an area under the curve of 0.98, 95% CI: 0.90–1 (Supplementary Figure S11).

Regarding the evaluation of the ^1^H-NMR aromatic region of the CPMG spectra, the samples were clustered within osteosarcoma or healthy control in the PCs 1, 2, and 5, which explained 24.5% of the variance (Supplementary Figure S12). The PLS-DA model classified the samples achieving 94.5, 97.6, and 84.6% of accuracy, sensitivity, and specificity, respectively, by LOOCV (Supplementary Figure S13). The metabolites with higher contributions to the discrimination were histidine, phenylalanine, and tyrosine (Figure 5, Supplementary Table S1 and Supplementary Figure S10). In the univariate analysis, only phenylalanine was statistically different (Supplementary Figure S9).

An exploratory analysis only in patients with osteosarcoma indicated a clustering tendency of samples according to the presence or absence of metastasis and according to the age (Supplementary Figure S14). The metastasis clusters are observed on PC 1 scores, which explained 31.8% of the variance of the samples, and the loadings indicated a relation with the samples' lipids amounts (Supplementary Figure S15). The differences related to the diagnosis age may be related to several metabolites, including glucose, alanine, and lactate (Supplementary Figure S15), in PC 9, explaining 2.54% of the samples' variance. However, the small number of cases and the mismatches of the classes preclude any conclusions.

## Discussion

An untargeted metabonomics analysis by ^1^H-NMR on blood serum samples from patients with osteosarcoma without any prior anticancer treatment showed that the disease resulted in metabolic alterations compared with healthy individuals. The high values of accuracy, sensitivity, and specificity validate the predictive capacity of the models. Based on these models, several discriminatory metabolites were identified, many of which are linked to energy metabolism [lipids, amino acids, and tricarboxylic acid (TCA) cycle]. Amino acids such as Gln, Ala, and Leu were identified too, and these compounds were already reported as altered in cancer metabolism. Amino acids, FAs, and glucose are used for energy balance and can change dynamically throughout tumorigenesis (8). These metabolites are linked to multiple pathways in cancer metabolisms, such as anaplerotic reactions for the TCA cycle providing an alternative energy source for cancer cells, which predominantly use energy produced by glycolysis rather than the TCA cycle and oxidative phosphorylation (22). Valine also contributes to bioenergetics and, besides having a role in protein synthesis, is one of the major nitrogen donors for Ala and Gln syntheses; in addition, substrates for the TCA cycle are obtained from valine degradation (23).

Interestingly, aromatic amino acids, such as Phe, Tyr, and His, were also increased in patients with osteosarcoma. An increased need for specific amino acids is observed in several cancers, requiring exogenous supply or causing upregulated *de novo* synthesis. In human osteosarcoma cancer stem cells, the amino acid demand is usually higher to counterbalance the decrease in the TCA cycle and undergo other physiological activities (24). These changes could be related to the alterations observed in the lipid metabolism, considering that they are essential for cell proliferation and energy source.

Lipoproteins (LPs) constitute a means of transport and recirculating reservoir for lipids (25, 26); these lipids are mainly triglycerides and cholesterol (Cho) or Cho esters. Due to the esters' reduced mobility, ^1^H-NMR signals of their methyl (-CH_3_), at 0.80 ppm, and methylene (-CH_2_-) hydrogen atoms, at 1.20 ppm, are broadened, which were found more elevated in osteosarcoma patients. The methyl (-CH_3_) hydrogen atoms of larger LP particles and with lower density [i.e., very-low-density lipoprotein (VLDL) and low-density lipoprotein (LDL)] present signals in higher frequency than smaller LPs (i.e., high-density lipoprotein) (26).

Cho is an important constituent of LP fractions, including LDL and VLDL, but 75% of total Cho is transported as LDL. The cells can catch and maintain this type of Cho; however, this fraction of Cho is more susceptible to oxidation by ROS, resulting in lipid peroxidation. To obtain enough Cho, proliferating cells can increase the rate of Cho biosynthesis by activating oncogenes that can transform the cells and activate anabolic and biosynthetic pathways (27), normally increasing the glycolytic pathway to produce energy, and transporting citrate for lipid biosynthesis (28). Associations between Cho and FAs such as elaidic acid, octadecanoic acid, and docosahexaenoic acid were already reported for lung metastasis in mouse models (29).

Elevated concentrations of LPs in the blood (hyperlipidemia) could be used as a marker of increased risk of relapse or tumor progression. In tumors like leukemia, it was already reported an association of hyperlipidemia with the accumulation of Cho (30), which were noted through histological examination and lipid droplet staining (31).

Exploring targeted sequencing data of osteosarcoma tumor samples from the same cohort of patients here studied (data not shown/published; methodology in Supplemental Material), we looked for variants mapped to 40 out 50 Cho genes (according to the Kyoto Encyclopedia of Genes and Genome; Supplementary Figure S16) that were represented in the gene panel. Mutations were detected in six genes (*PLTP, ABCB11, APOB, SORT1, LRP2*, and *ABCG8*), all of them missense variants (Supplementary Table S3); mutations in *LRP2, ABCG8*, and *PLTP* were not considered for further discussion because they exhibited low allelic frequency (<10% of the reads). *APOB* codifies for a protein component of LDL and VLDL-Cho; the quantity of APOB indicates the amount of Cho and, consequently, of potentially atherogenic LPs (32). Actually, the LPs are used, such as nanotechnology-based drug delivery vehicles or imaging agents to desired sites (33). Additionally, studies indicated that *PLTP* promotes the transfer of several lipid molecules (34, 35), influencing lipid and LP metabolism. Serum lipids and LP levels exhibit a notable rise until the age of 2 years and during sexual maturation. Also, the same goes for LDL-Cho in puberty (36).

Therefore, we hypothesized that osteosarcomas, similar to clear-cell carcinoma cells (37), may use the triglycerides for energy consumption, accumulating cholesteryl esters by increased acyl-CoA/Cho acyltransferase (*ACAT*) activity. These might point to lipid accumulation that partially depends on an increase in the uptake of plasma LPs, possibly by hypoxia-inducible factor (*HIF1A*) mediation that induced overexpression of very-low-density receptor *VLDLR* (37) or through an alternative receptor. In agreement with this hypothesis, several osteosarcoma cell lines present a high expression of *ACAT, HIF1A*, and *VLDLR* (Supplementary Table S4).

The cohort used in this study was selected according to the study design and samples' availability, and it called attention because 44% of the patients had metastasis at diagnosis. This higher number of metastasis at diagnosis was previously reported for Brazilian patients with osteosarcoma, which also present a more aggressive pattern (38). In general, patients treated in the Barretos Cancer Hospital (Hospital de Câncer de Barretos) may not reflect the Brazilian population because it is a highly specialized cancer center with patients who are referred from many regions that lack resources, which hinders early detection and appropriate diagnosis (39); at the moment of hospitalization when the medical care was asked for the first time and nor rarely, months can pass before a diagnosis of osteosarcoma.

As our study presents some limitations, such as the small number of investigated patients as well as age and sex mismatched controls, additional proof-of-concept studies must be carried out. Still, in an untargeted ^1^H-NMR metabonomic approach, blood serum osteosarcoma patients were investigated with great success. Increases in metabolites linked with lipid metabolism pathways and amino acid biosynthesis were detected. Therefore, the reported findings raise the possibility of using blood biomarkers as a tool that could add-in to accurate diagnosis of osteosarcoma, as well as, a better follow-up of the disease.

## Data Availability Statement

All datasets generated for this study can be provided if contacted the authors.

## Ethics Statement

The studies involving human participants were reviewed and approved by Barretos Cancer Hospital Ethical Committee (CEP-HCB 898.403) and from the Institute of Biosciences of the University of São Paulo (controls CEP-IBUSP-2589398). Written informed consent to participate in this study was provided by the participants' legal guardian/next of kin.

## Author Contributions

MQ, MM, and LT designed the research. MQ and LM performed the NMR experiments. TC performed the chemometrics analysis. SC performed targeted sequencing experiments. SC performed the next-generation sequencing experiments, and AK supervised and analyzed the target sequencing data of tumors, as well as collected control samples. AL, ÉB, LL, SM, and DV selected the cases, collected and provided osteosarcoma samples, and revised and provided clinical data of osteosarcoma patients. MQ and TC analyzed the experimental data. LT provided reagents and supervised metabonomic and NMR experiments. MM provided reagents and supervised the study. MQ, TC, MM, and LT wrote the paper. All authors read and approved the final manuscript.

## Conflict of Interest

The authors declare that the research was conducted in the absence of any commercial or financial relationships that could be construed as a potential conflict of interest.
