# Supplementary material for: Insights in Osteosarcoma by Proton Nuclear Magnetic Resonance Serum Metabonomics
Source: Front Oncol. 2020 Oct 16;10:506959. doi: 10.3389/fonc.2020.506959 (PMC7596414; doi:10.3389/fonc.2020.506959)
Supplement: Supplementary file 1 [file Data_Sheet_1.PDF]

Supplementary material

Insights in Osteosarcoma by ^{1}H-NMR serum metabonomics

Melissa Quintero Escobar[1]+, Tassia Brena Barroso Carneiro Costa[1]+, Lucas G. Martins[2], Silvia S. Costa[3], André van-Helvoort Lengert[4], Érica Boldrini[5], Sandra Regina Morini da Silva[6], Luiz Fernando Lopes[5], Daniel Onofre Vidal[4], Ana C. Krepischi[3], Mariana Maschietto[7], and Ljubica Tasic[1*].

[1] University of Campinas (UNICAMP), Institute of Chemistry, Department of Organic Chemistry, Campinas (SP), Brazil, [2] Facultad de Ingeniería Industrial, Universidad de Lima, Peru, [3] University of Sao Paulo (USP), Institute of Biosciences, Department of Genetics and Evolutionary Biology, Human Genome and Stem-Cell Research Center (CEGH-CEL), Sao Paulo (SP), Brazil, [4] Barretos Cancer Hospital, Molecular Oncology Research Center (CPOM), Barretos (SP), Brazil, [5] Barretos Children's Cancer Hospital, Barretos, SP, Brazil, [6] Barretos Cancer Hospital, Department of Pathology, Barretos, SP, Brazil, [7] Brazilian Biosciences National Laboratory (LNBio), Brazilian Center for Research in Energy and Materials (CNPEM), Campinas, SP, Brazil

+Both authors contributed equally to this work
*Correspondence:
Prof. Ljubica Tasic, Ph. D. E-mail: ljubica@unicamp.br

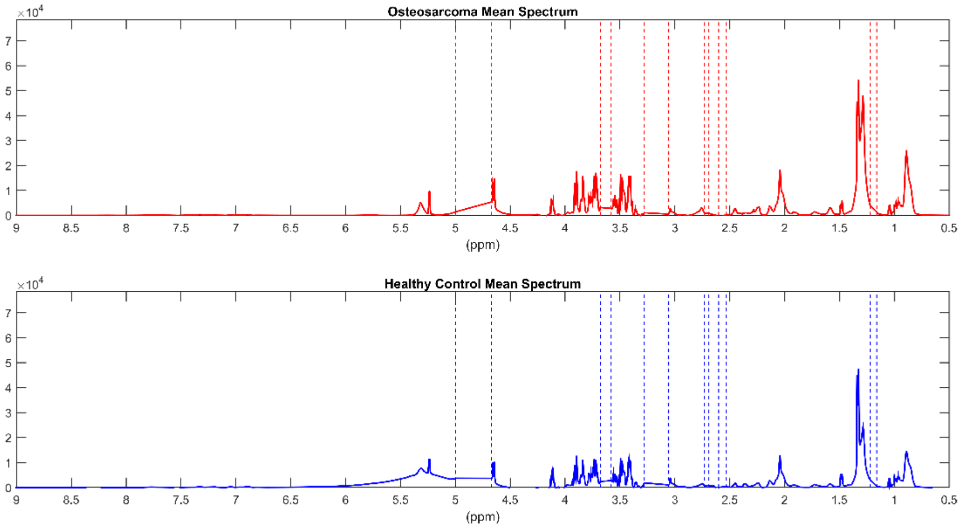

Figure S1. ^{1}H-NMR mean spectra of healthy control (in blue - upper panel) and osteosarcoma patients (in red - lower panel) acquired using CPMG pulse sequence. The HDO region from δ 4.70–5.00, the EDTA (δ 2.53–2.73, δ 3.06–3.28, δ 3.6–3.65) and the ethanol (δ 1.16 – 1.22, δ 3.58 – 3.68), were removed before analyses and are indicated with the dotted lines.

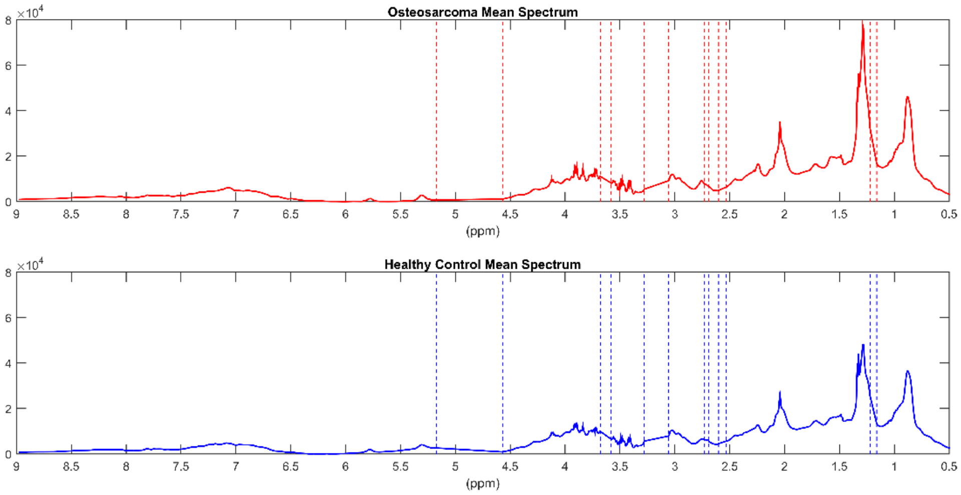

Figure S2. ^{1}H-NMR mean spectra of healthy control (in blue - upper panel) and osteosarcoma patients (in red - lower panel) acquired using Watergate pulse sequence. The HDO region from δ 4.70–5.00, the EDTA (δ 2.53–2.73, δ 3.06–3.28, δ 3.6–3.65) and the ethanol (δ 1.16 – 1.22, δ 3.58 – 3.68), were removed before analyses and are indicated with the dotted lines.

Table S1. ^{1}H-NMR chemical shifts assignments of the metabolites found in osteosarcoma and healthy controls blood serum samples where s, singlet; d, doublet; t, triplet; q, quartet; m, multiplet; dd, doublet of doublets. Metabolites with great importance according to variable importance in projection (VIP) are shown in red. Concentration differences in OS in comparison to HC are also shown.

Metabolites	Moieties	δ ^{1}H and multiplicity [a]
Fatty acyls	CH_3, -$(CH_2)_n$, CH_2CH_2CO, CH_2CH=, HC=CH	0.85-0.89 (m), 1.24-1.37 (m), 1.55-1.65 (m), 1.98-2.09 (m), 5.29-5.43 (m)
Valine	γCH_3, βCH, αCH	0.99 (d), 1.04 d), 3.61 (d)
Lactate	βCH_3, αCH	1.33 (d), 4.11 (q)
Alanine	βCH_3, αCH	1.48 (d), 3.77 (q)
Glutamine	γCH_2	2.12 (m), 2.45 (m), 3.77 (t)
Glucose	C1H, C2H, C3H, C4H, C5H,1/2 CH_2-O6	3.24 (dd), 3.39 (m), 3.46 (m), 3.53 (dd), 3.72 (m), 3.84 (m), 3.89 (dd), 4.65 (d), 5.24 (d)
Tyrosine	CH, CH	6.89 (m), 7.19 (m)
Histidine	4CH, CH_2	7.05 (s), 7.75 (s)
Phenylalanine	βCH_2, β'CH_2, αCH, 2 or 6 CH, 4 CH, 3 or 5 CH	7.33 (d), 7.37 (m), 7.42 (m)
Formate	CH	8.46 (s)

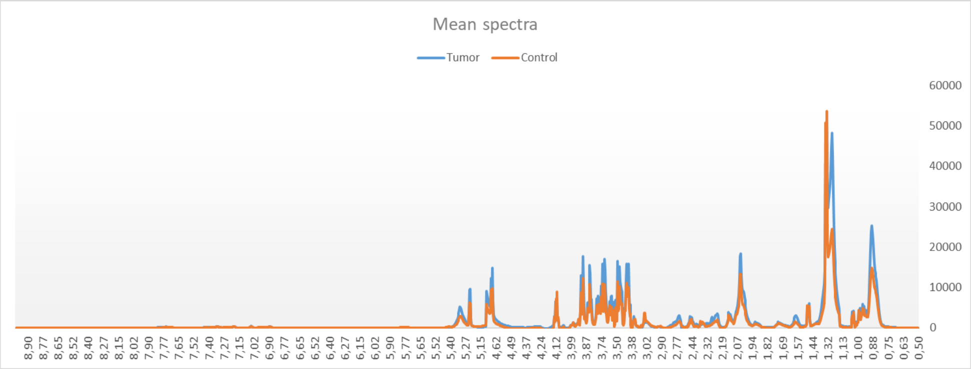

Lipids	HC: 2.2 mmol L^{-1}<br>OS: 13.8% increase	Tyrosine	HC: 0.07 mmol L^{-1}<br>OS: 5.5% increase
Lactate	HC: 2.2 mmol L^{-1}<br>OS: 11.6% decrease	Histidine	HC: 0.6 mmol L^{-1}<br>OS: 4% increase
Glucose	HC: 4.5 mmol L^{-1}<br>OS: 12% increase	Phenylalanine	HC: 0.8 mmol L^{-1}<br>OS: 6.2% increase

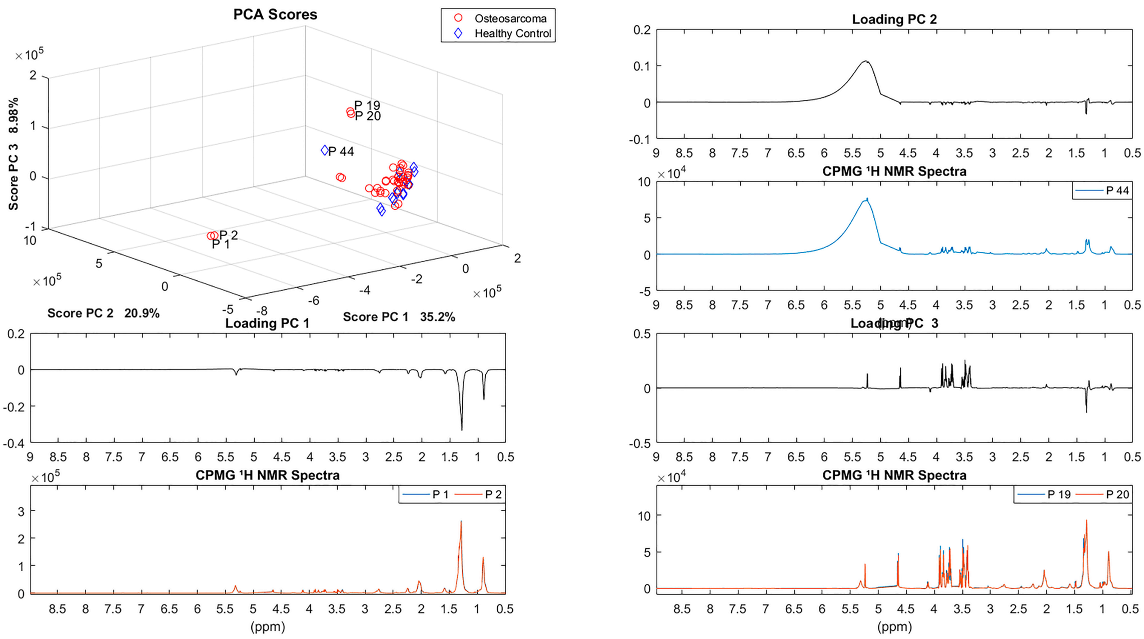

Figure S3. Identification of outliers by principal components analysis (PCA) applied on ^{1}H-NMR CPMG spectra: scores on the first three principal components; loading plots of each principal component and the corresponding outlier's spectra. Osteosarcoma patients are in red circles and Healthy controls are in blue diamonds.

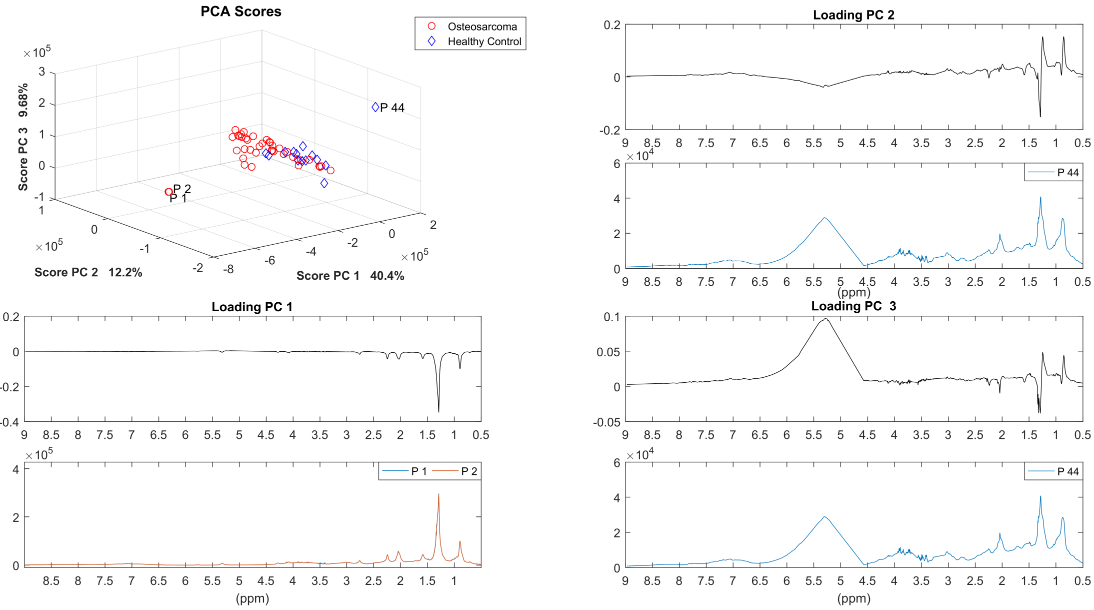

Figure S4. Identification of outliers by principal components analysis (PCA) of ^{1}H-NMR Watergate spectra: scores on the first three principal components; loadings of each principal component and the corresponding outlier's spectra. Osteosarcoma patients are in red circles and Healthy controls are in blue diamonds.

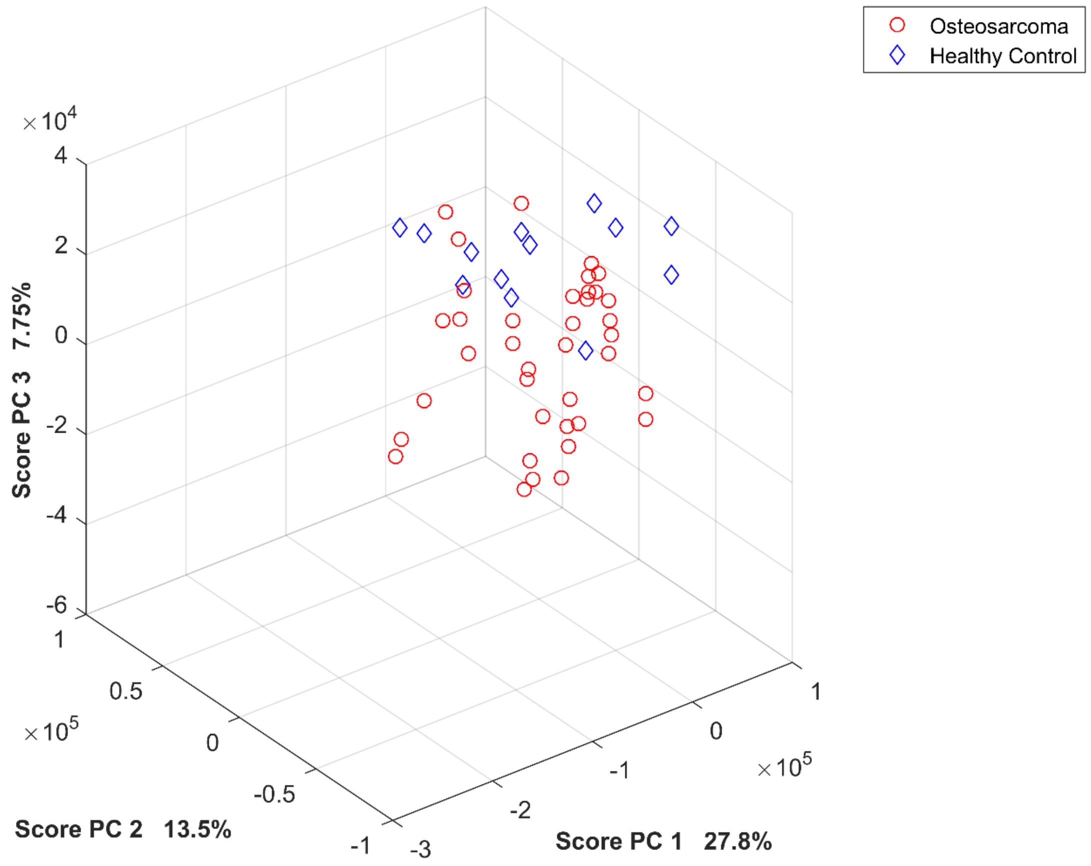

Figure S5. Scores on the first three principal components obtained from the ^{1}H-NMR CPMG spectra. Osteosarcoma patients are in red circles and Healthy controls are in blue diamonds.

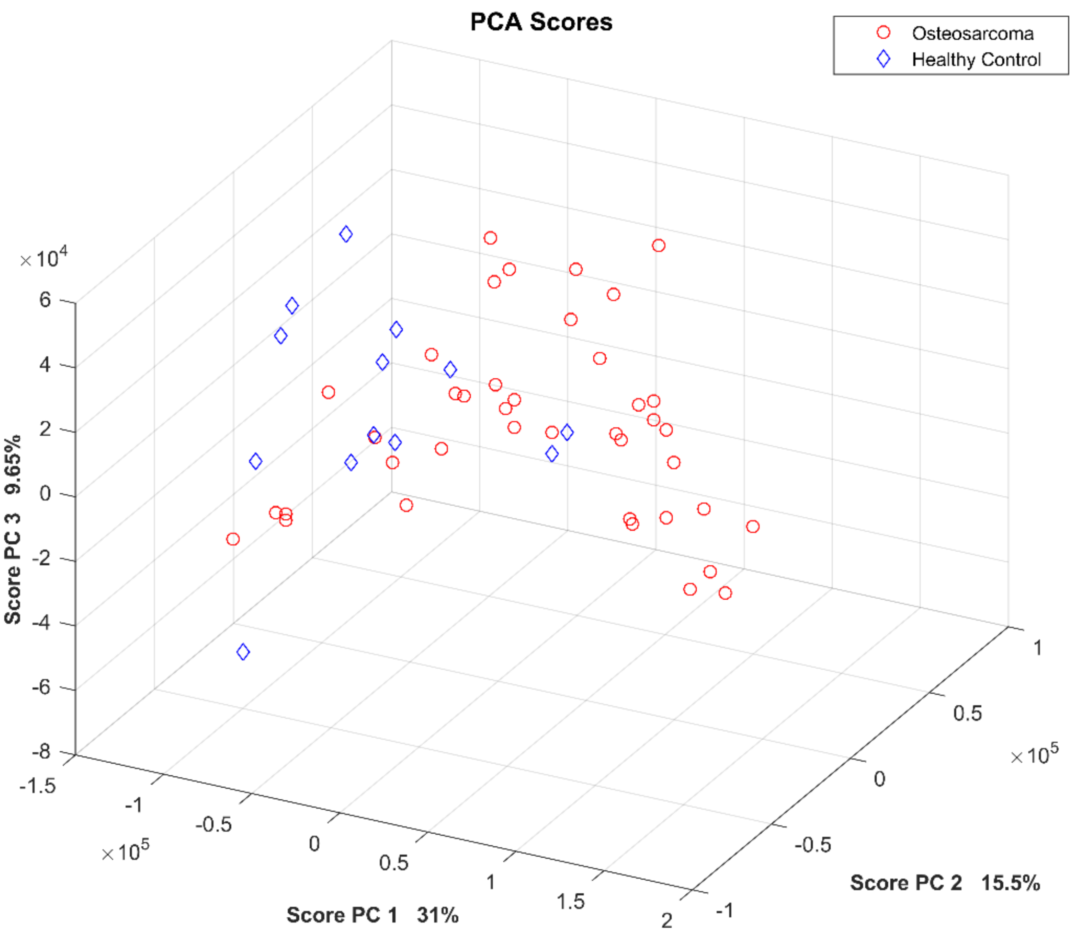

Figure S6. Scores plot on the first three principal components obtained from the ^{1}H-NMR Watergate spectra. Osteosarcoma patients are in red circles and Healthy controls are in blue diamonds.

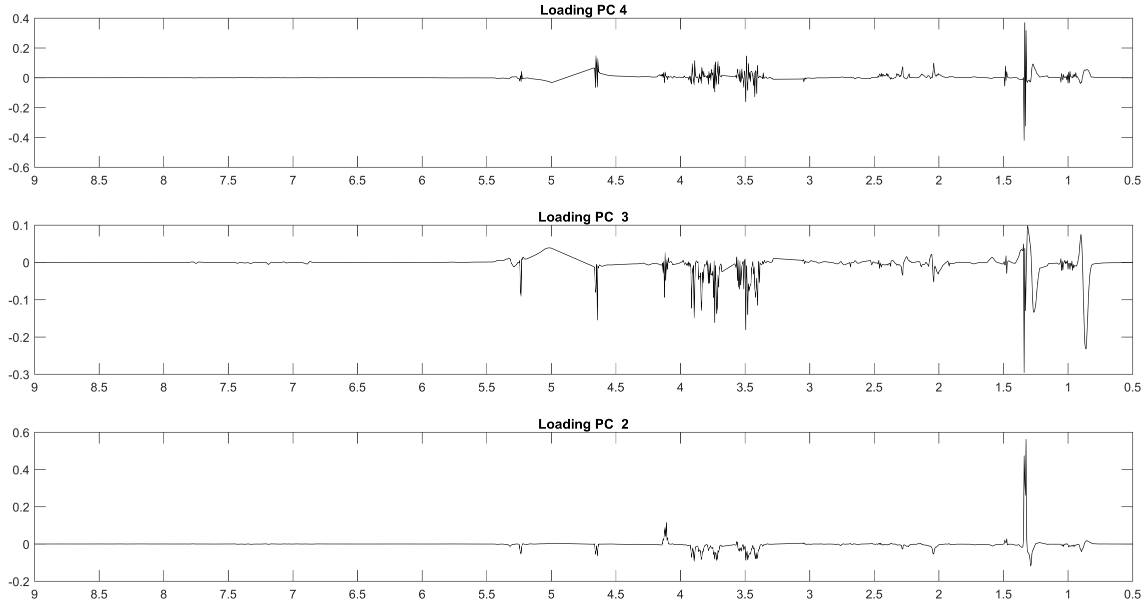

Figure S7. Loadings of the principal components 2, 3 and 4, obtained from the ^{1}H-NMR CPMG spectra.

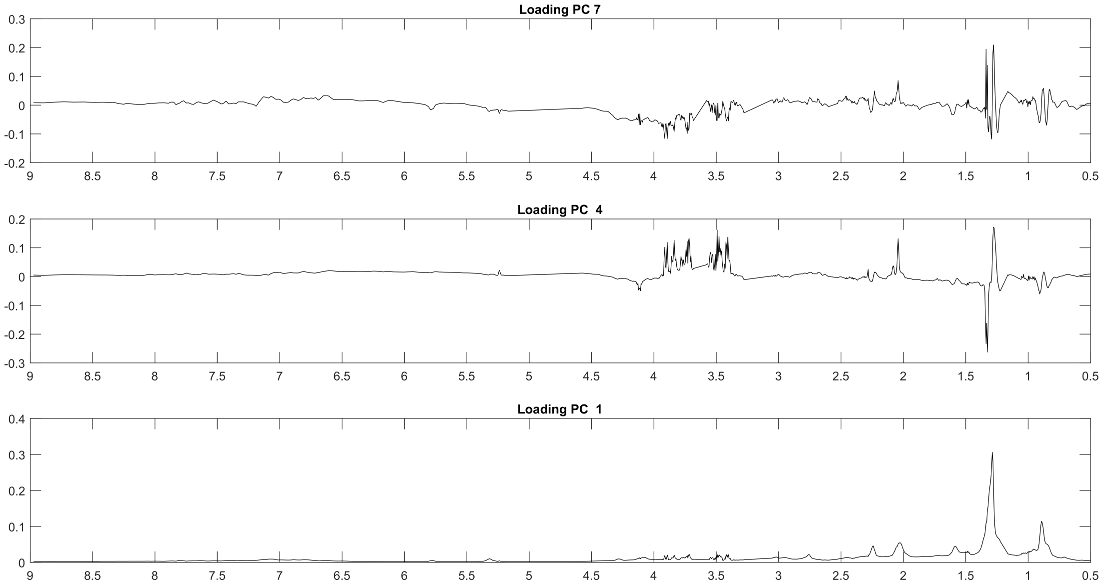

Figure S8. Loadings of the principal components 1, 4 and 7, obtained from the ^{1}H-NMR Watergate spectra.

Table S2. Classification parameters of PLS-DA models after LOOCV.

Parameters	CPMG	Watergate
Sensitivity	92.1	95.0
Specificity	92.3	92.3
Accuracy	92.2	94.3

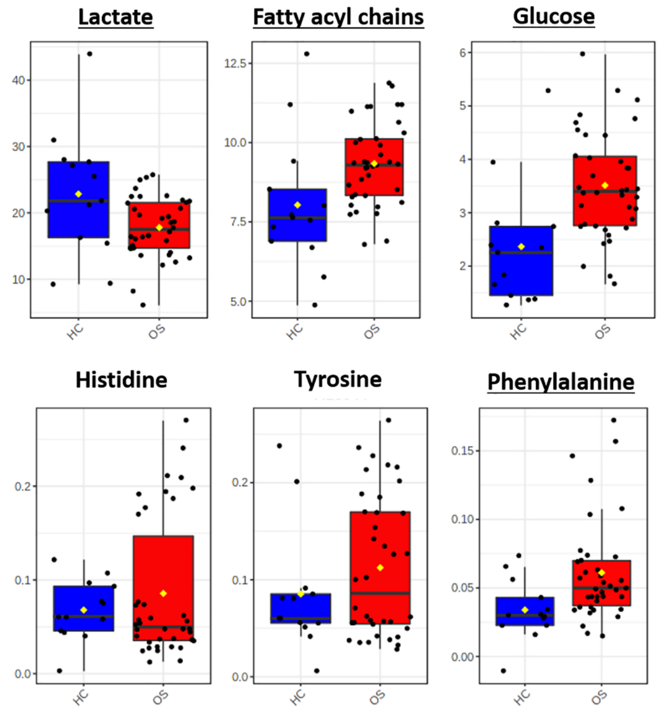

Figure S9. Box plots representing the variations of concentrations of metabolites indicated as discriminant by PLS-DA (VIP scores > 2). Underlined presented a statistically significant difference between osteosarcoma patients and healthy controls. The black dots represent the concentrations from all samples, and the yellow diamond is the mean. HC showed the common, considered healthy metabolites' concentrations that follow in blue while the increase or decrease of the same metabolites are given in % in red: 1) lactate 2.2 mmol L^{-1} (decrease of 11.6% in OS) 2) lipids (fatty acyl hydrogens): 2.2 mmol L^{-1} (increase of 13.8% in OS) 3) glucose: 4.5 mmol L^{-1} (increase of 12% in OS) and 4) histidine: 0.6 mmol L^{-1} (increase of 4% in OS) 5) tyrosine: 0.07 mmol L^{-1} (increase of 5.5% in OS) and 6) phenylalanine: 0.8 mmol L^{-1} (increase of 6.2% in OS).

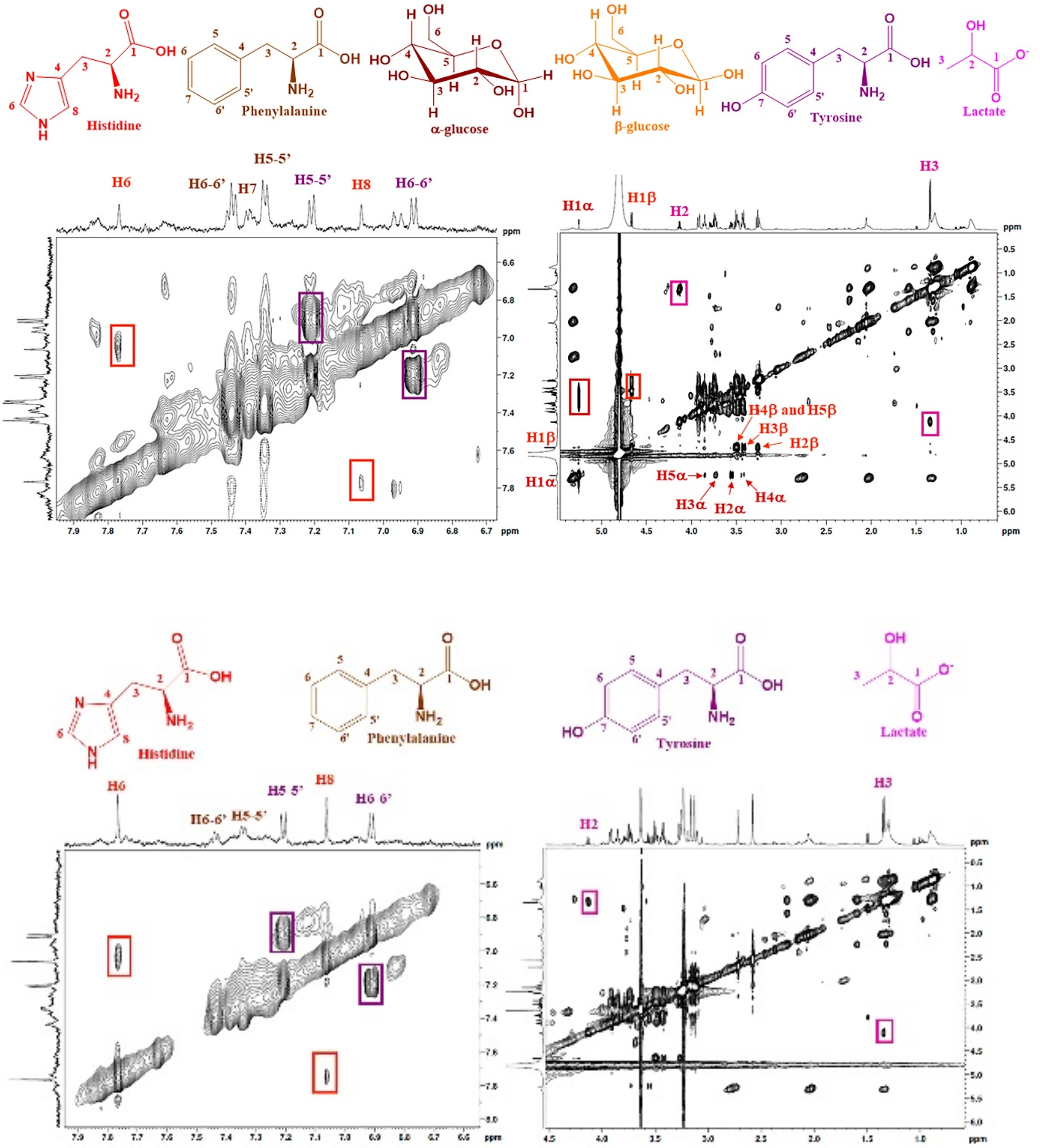

Figure S10. TOtal Correlation SpectroscopY (TOCSY) map obtained for the representative samples of each group with metabolites assignments. Osteosarcoma patient (upper panel) and Healthy control (lower panel).

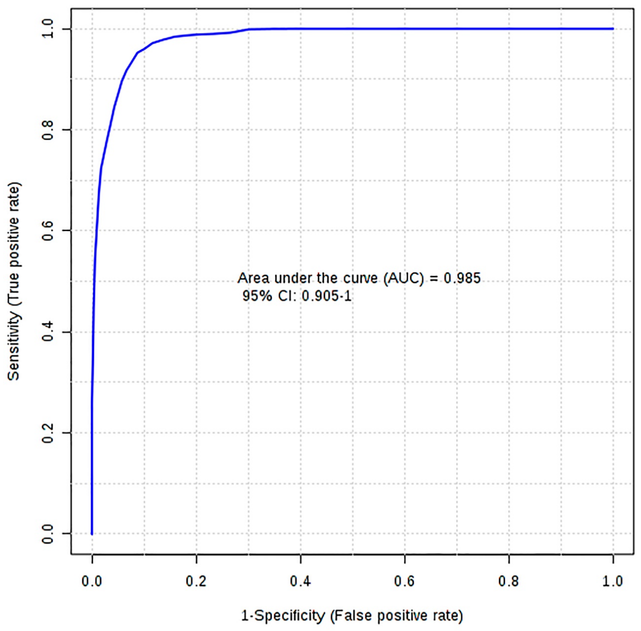

Figure S11. ROC curve for the differential metabolites model based upon its average performance across all MCCV runs.

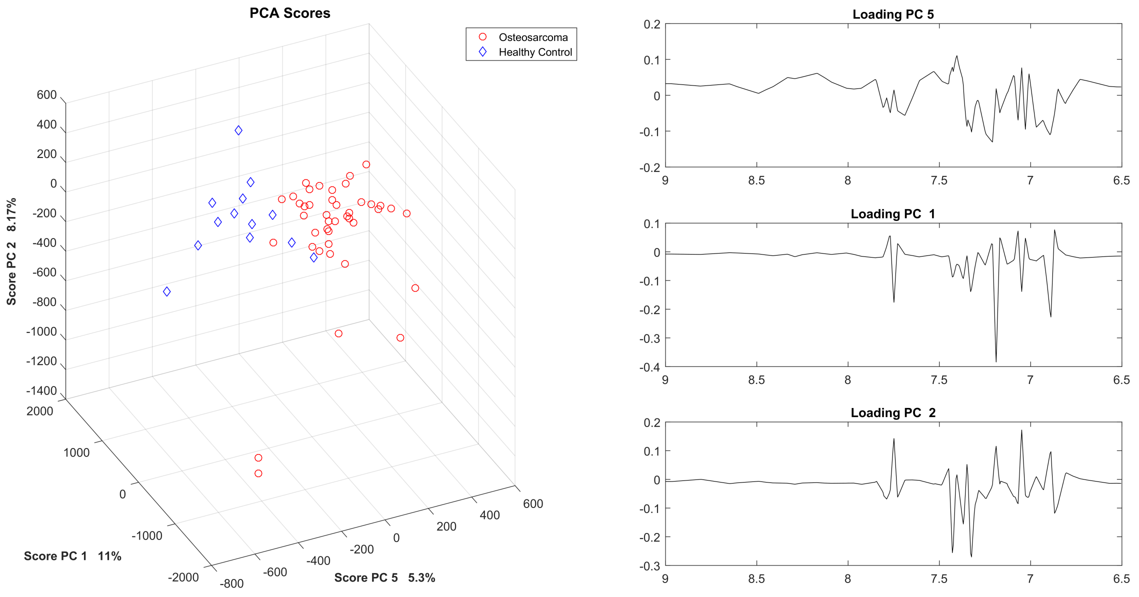

Figure S12. Scores and loadings of PCs 1, 2, and 5 of the ^{1}H-NMR CPMG aromatic region. Osteosarcoma patients are in red circles and the healthy controls are in blue diamond.

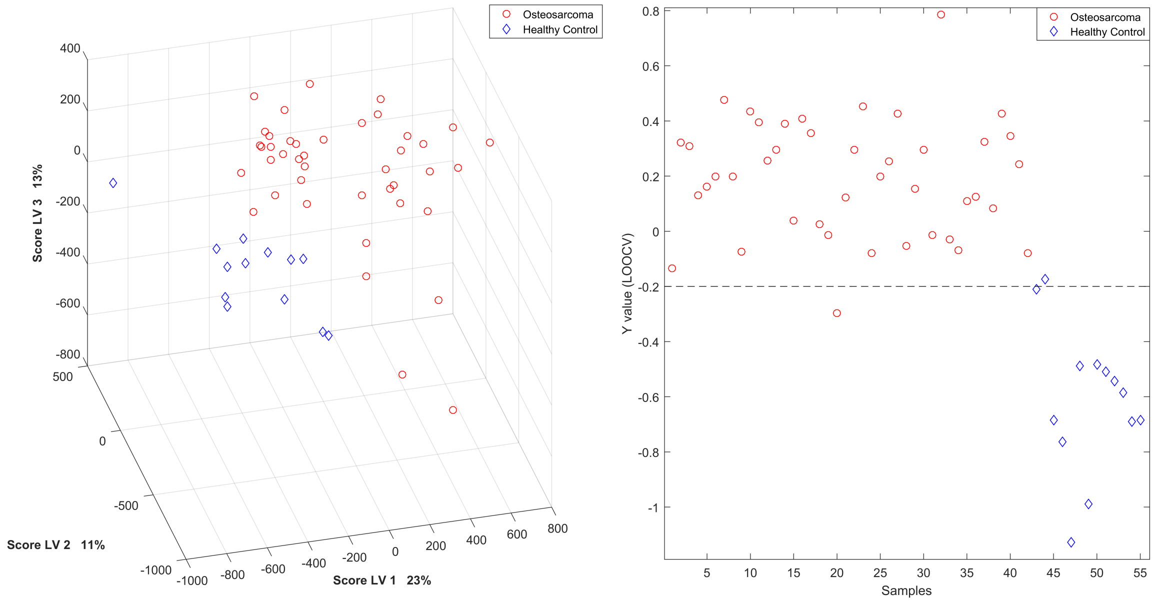

Figure S13. PLS-DA scores plot and Y calculated for each sample, by LOOCV, of the ^{1}H-NMR aromatic region CPMG spectra model. Osteosarcoma patients are in red circles and the healthy controls are in blue diamond.

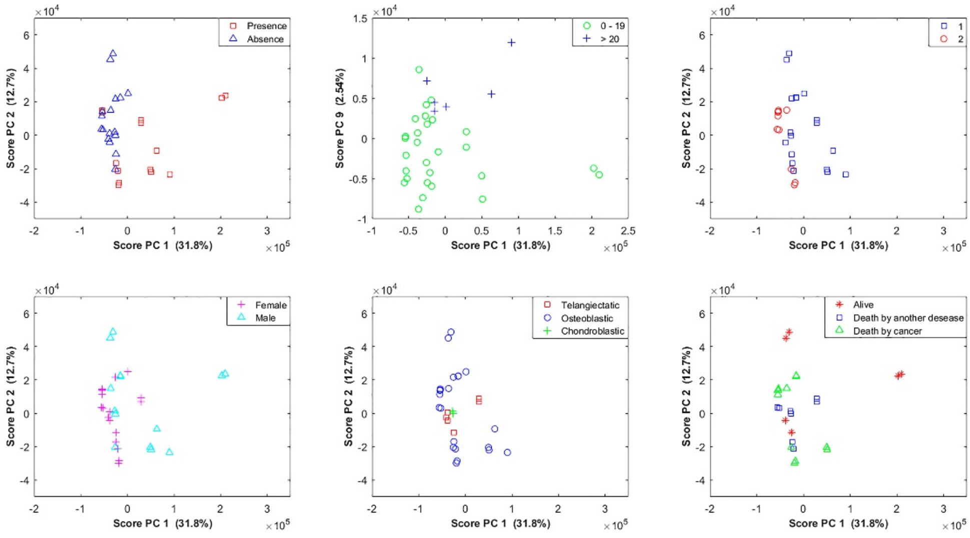

Figure S14. ^{1}H NMR CPMG score-graphs of PCA related to the clinical characteristics of the patients. Exploratory analysis inside the osteosarcoma patients group according to metastasis at diagnosis (presence or absence), age (0-19 and older than 20), Huvos grade (1 or 2), sex (female and male), histology type (telangiectatic, osteoblastic and chondroblastic), and status characteristics (alive, death by another disease or death by cancer).

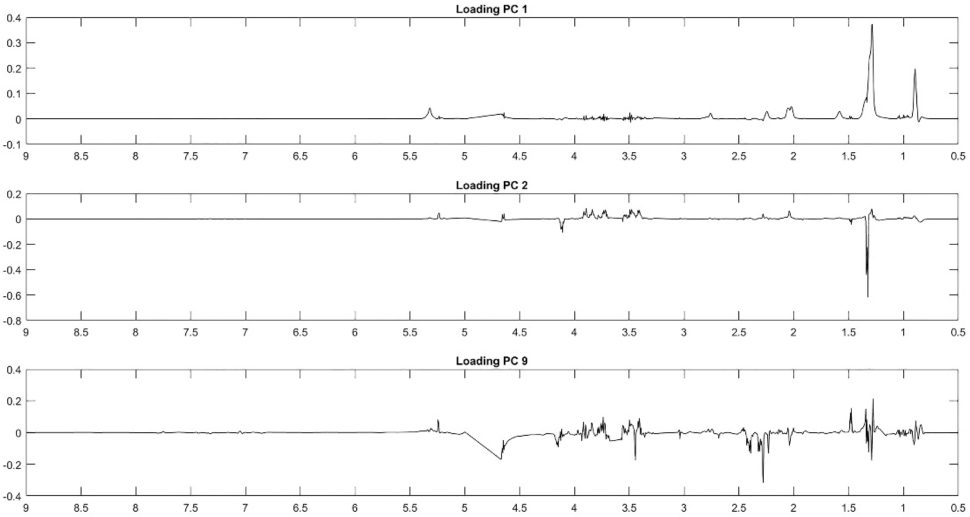

Figure S15. Loadings of the principal components (PCs) 1, 2 and 9 of the model built from the patients CPMG spectra.

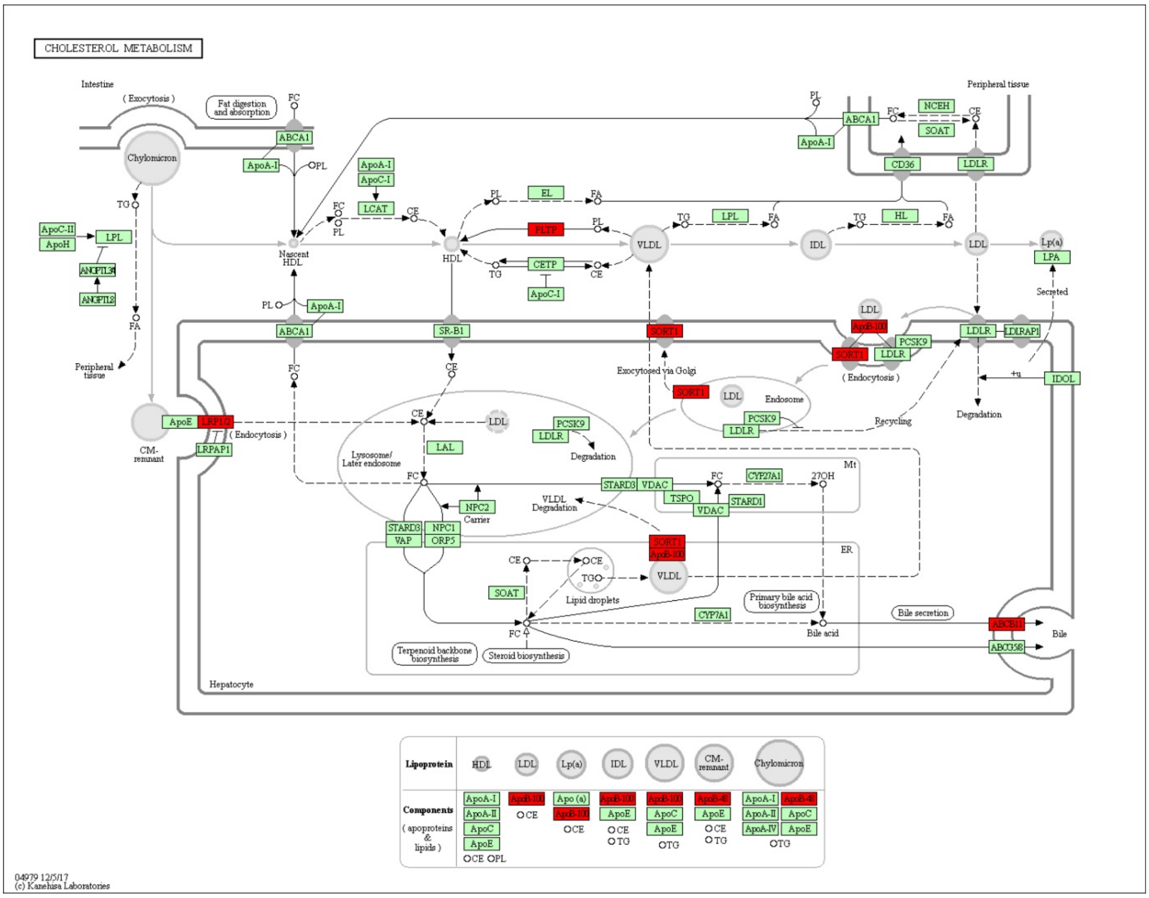

Figure S16. Kyoto Encyclopedia of Genes and Genomes (KEGG) Cholesterol pathway. In red the genes (PLTP, ABCB11, APOB, SORT1, LRP2 and ABCG8) mutated in the tumors from osteosarcoma patients.

Table S3. Gene mutations founded in osteosarcoma patients. *ABCG8: ATP-binding cassette sub-family G member 8; ABCB11: ATP Binding Cassette Subfamily B Member 11; APOB: apolipoprotein; LRP2: low density lipoprotein-related protein 2; PLTP: phospholipid transfer protein; SORT1: sortilin 1.*

Gene Mutation	Chr:Pos	Ref/Alt	Var Allele Freq
↓*LRP2*	2:170099931	A/T	0.02
PLTP/SORT1	20:44528480 /1:109940370	G/T : G/C	0.79 / 1.00
↓*ABCG8*	2:44078903	C/A	0.02
APOB	2:21235118	G/A	0.53
↓*PLTP*	20:44528480	G/T	0.02
ABCB11	2:169781238	C/T	0.56

Table S4. Gene expression data of *ACAT*, *VLDLR* and *HIF1A.* GE in osteosarcoma cell lines obtained from the expression atlas from the European Molecular Biology Laboratory and the European Bioinformatics Institute (EMBL-EBI). *Cut off 0.5 TPM

Gene ID	Gene Name	143b	CAL-72	HAL	HOSMNNG	HuO-3N1	HuO9	KPD	MHM	NOS-1	NY	OHS	OST	Sarc9371
ENSG00000075239	ACAT1	62.0	81.0	62.0	40.0	19.0	65.0	27.0	16.0	56.0	20.0	76.0	60.0	21.0
ENSG00000120437	ACAT2	65.0	119.0	67.0	89.0	31.0	100.0	27.0	23.0	18.0	51.0	36.0	35.0	91.0
ENSG00000147852	VLDLR	14.0	6.0	24.0	2.0	6.0	43.0	6.0	8.0	23.0	13.0	3.0	15.0	9.0
ENSG00000100644	HIF1A	167.0	231.0	182.0	209.0	812.0	232.0	101.0	282.0	270.0	341.0	114.0	941.0	343.0

DNA sequencing

For DNA sequencing, tumor samples were reviewed by a pathologist prior to microdissection to select areas with at least 70% of cells of interest. DNA was isolated in a QIA symphony equipment with the DSP DNA Mini Kit (Qiagen). Quantity and quality were assessed by NanoDrop (Thermo Fisher Scientific) and agarose gel 0.8%.

Targeted sequencing of tumors

Genomic libraries of 21 osteosarcoma samples were enriched using the TruSight One Sequencing Panel Kit (Illumina, USA), composed by 4,812 genes of clinical relevance. The libraries were paired end sequenced on a HiSeq 2500 sequencing platform (Illumina, San

Diego, USA). BWA files were aligned with the genome of reference (hg19/Grch37) using the Burrows-Wheeler Aligner (BWA) package version 0.6.1, and local realignment was carried out with the Genome Analysis Tool Kit (GATK) version 1.6. Variant annotation and filtering from VCF files were run using VarSeq software (Golden Helix, Bozeman, USA).

Using the Kyoto encyclopedia of genes and genome KEGG, we retrieved 50 genes belonging to the cholesterol metabolism. Exploring targeted sequencing data from osteosarcoma samples from the same cohort of patients here studied (data not published), we looked for variants mapped to 40 out 50 cholesterol genes that were represented in the panel. This study was based on the selection of coding variants presenting read depth > 20 reads, Phred scores for a variant confidence above 20, at least 10% of variant allele frequency in the sample, and resulting in missense or loss-of-function (essential splice site, frameshift or gain/loss of stop-codons). Only missense variants evaluated as damaging to the protein function were retained, according to *in silico* algorithms (PolyPhen-2, http://genetics.bwh.harvard.edu/pph2/; SIFT, http://sift.jcvi.org/; MutationTaster, http://www.mutationtaster.org/). Germline variants deposited in public databases were excluded (http://www.1000genomes.org/; http://www.ncbi.nlm.nih.gov/projects/SNP/; http://exac.broadinstitute.org/; http://gnomad.broadinstitute.org/), including AbraOM, which contains exome data of 607 healthy Brazilians (DOI:10.1002/humu.23220).
